# Remarkable Antitumor Effects and Serious Multiple Immune-Related Adverse Events in Malignant Pleural Mesothelioma: Two Case Reports

**DOI:** 10.1155/crom/8768823

**Published:** 2025-03-03

**Authors:** Koharu Harada, Hidehiro Irie, Akifumi Mitsuishi, Takahiro Fukui, Nao Takada, Ryosuke Nagaoka, Yohei Funatsu, Hidefumi Koh

**Affiliations:** ^1^Division of Pulmonary Medicine, Department of Internal Medicine, Tachikawa Hospital, Tachikawa, Tokyo, Japan; ^2^Division of Infectious Diseases and Respiratory Medicine, Department of Internal Medicine, National Defense Medical College, Tokorozawa, Saitama, Japan

**Keywords:** antitumor effect, immune-related adverse events, ipilimumab, malignant pleural mesothelioma, nivolumab

## Abstract

We describe two patients who experienced serious multiple immune-related adverse events (irAEs), treatment interruption, and steroid administration. Despite these challenges, they achieved a remarkable antitumor effect beyond the expected. Various carcinomas demonstrated a possible correlation between the antitumor effect of immune checkpoint inhibitors and the intensity of irAEs, but few studies report on malignant pleural mesothelioma (MPM). Our two cases exhibited much stronger irAEs than usual. These two cases still demonstrated a complete response (CR) or near CR partial response, indicating a correlation between irAEs and the antitumor effect in MPM.

## 1. Introduction

Malignant pleural mesothelioma (MPM) is an extremely aggressive disease with a poor prognosis. Patients with MPM are frequently diagnosed during an unresectable stage, emphasizing the importance of effective medical therapy. Despite the low prevalence of MPM, there have been slow but steady therapeutic advances in MPM. For example, adding bevacizumab to pemetrexed plus cisplatin was reported to improve OS in MPM significantly [1], making this regimen one of the first-line treatments for MPM [2]. Meanwhile, since the efficacy of immune checkpoint inhibitors (ICIs) in lung cancer treatment was approved, new ICI-based treatment regimens have dramatically changed lung cancer chemotherapy. As with lung cancer, the development of ICI-containing regimens for MPMs and the improvement of MPM prognosis with ICI treatment were expected.

The CheckMate 743 trial revealed the superiority of the combination therapy of ICIs with nivolumab plus ipilimumab in MPM, exhibiting better overall survival compared to the previous chemotherapy [3], and this therapy was newly approved as a first-line treatment. Conversely, ICIs show ICI-specific side effects, named immune-related adverse events (irAEs), which sometimes make chemotherapy continuation difficult or even cause a fatal outcome. However, some reports indicate that patients who experience stronger irAE may demonstrate more effective antitumor effects of ICIs [4, 5], and the association between irAE and ICI efficacy, particularly in MPM, remains unclear. Here, we report two cases of MPM demonstrating favorable antitumor efficacy with serious multiple irAEs with nivolumab plus ipilimumab.

## 2. Case Presentation

### 2.1. Case 1

**Figure 1:**
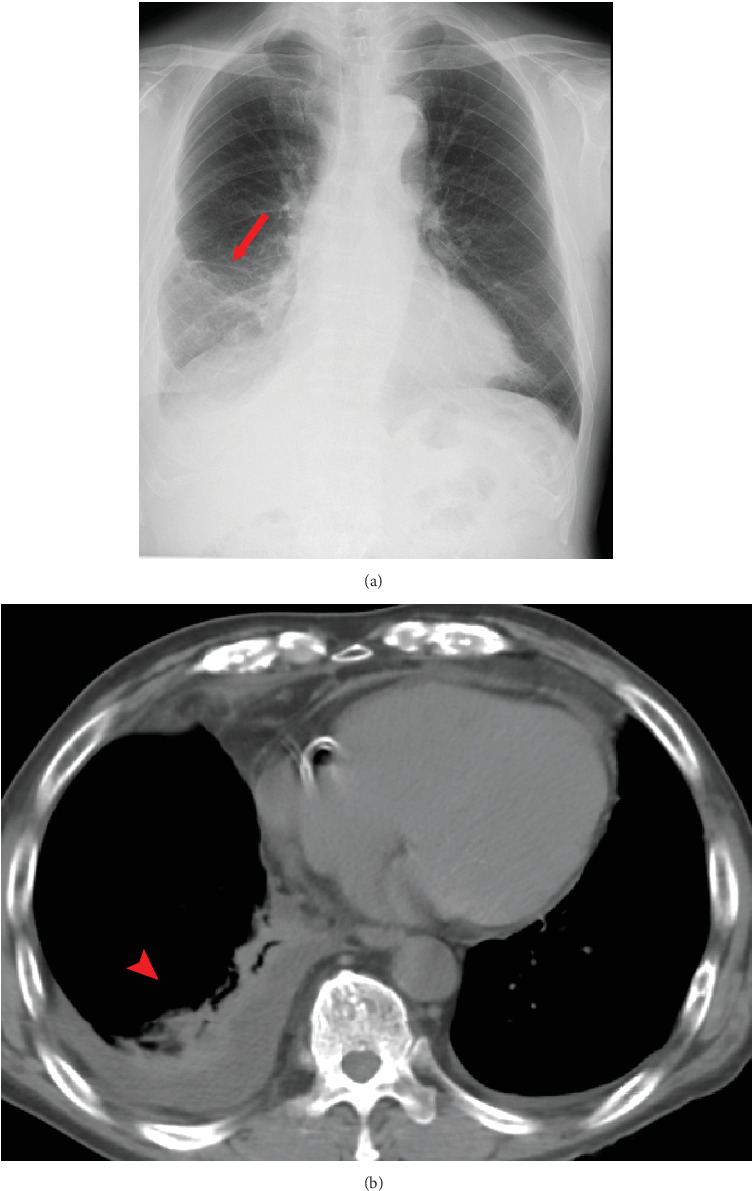
(a) Chest x-ray film and (b) CT image, revealing right pleural thickening, lung volume loss, and pleural effusion in Case 1. CT, computed tomography.

A 74-year-old male patient was referred with complaints of fever and dyspnea. The initial chest x-ray of the patient revealed a right-sided pleural effusion. Computed tomography (CT) scans revealed right pleural thickening, lung volume loss, and pleural effusion (Figure 1). Video-assisted thoracoscopic pleural biopsy was conducted, indicating the diagnosis of MPM (cT1N0M0 Stage I). First-line therapy with nivolumab (240 mg every 2 weeks) plus ipilimumab (1 mg/kg every 6 weeks) was initiated not only due to the surgical difficulty but also the patient's strong insistence on chemotherapy with ICI instead of surgery.

The patient developed diarrhea at the later period of the second treatment cycle, requiring second cycle interruption. The patient was identified with CTCAE irAE colitis (Grade 2), but his symptoms gradually improved without steroid administration, enabling him to resume the third cycle. However, diarrhea relapsed during the fourth cycle. The diarrhea was considered irAE colitis (Grade 3); thus, ICI therapy was discontinued, and oral prednisolone (PSL) of 0.5 mg/kg was initiated. Low-dose PSL was continued for over a month, which improved diarrhea symptoms. The patient insisted on resuming ICI chemotherapy; thus, the fifth treatment cycle was resumed. The patient complained of headaches during the sixth treatment cycle, and ICI therapy was reinterrupted. Head magnetic resonance imaging (MRI) revealed no significant abnormalities, but the lumbar puncture demonstrated increased spinal fluid monocytes. These test results ruled out bacterial meningitis and metastatic brain tumors. Based on the patient's general condition and these test results, we mainly suspected irAE meningitis or carcinomatous meningitis. Taking into consideration the choice of steroids for carcinomatous meningitis [6], we decided to use dexamethasone (DEX), and based on the use of high-dose steroids for irAE meningitis [7], we selected oral DEX 6 mg/day. After administration of DEX, the headache promptly improved. A few days later, cytology results of cerebrospinal fluid showed no malignant findings, and we diagnosed irAE meningitis (Grade 3).

As the headache improved, we started tapering the DEX dose, but the dose reduction of steroids caused new hepatic and renal dysfunction. The possibility of drug-induced hepatic and renal impairment was initially considered, but they did not improve after discontinuing the suspect drugs. The steroid dosage was increased (PSL 1 mg/kg), which improved liver and renal dysfunction, indicating irAE-related hepatic and renal impairment (hepatitis [Grade 3] and renal dysfunction [Grade 2]).

**Figure 2:**
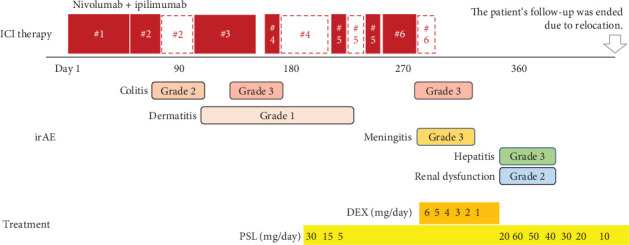
The summary of ICI therapy, irAE, and irAE-related treatment in Case 1. ICI, immune checkpoint inhibitor; irAE, immune-related adverse event; DEX, dexamethasone; PSL, prednisolone.

**Figure 3:**
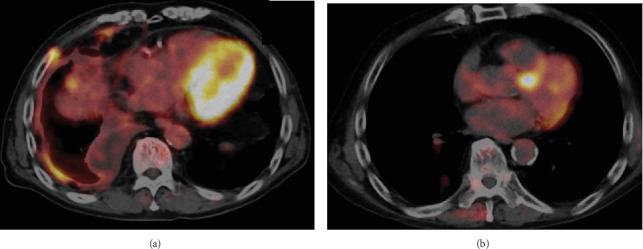
PET-CT scan (a) before ICI therapy and (b) after 1 year. Complete response (CR) was achieved. ICI, immune checkpoint inhibitor.

ICI therapy was repeatedly interrupted throughout these sixth-cycle periods, and steroids were required to control for multiple irAEs.  Figure 2 shows this process. However, a positron emission tomography-CT (PET-CT) scan taken 1 year after treatment initiation showed complete response (CR) despite frequent treatment interruptions and prolonged steroid administration (Figure 3). Based on the middle of six treatment courses, the patient was followed up without further chemotherapy due to the heavy irAE symptoms, but the patient experienced no recurrence within 20 months until follow-up was concluded due to relocation.

### 2.2. Case 2

**Figure 4:**
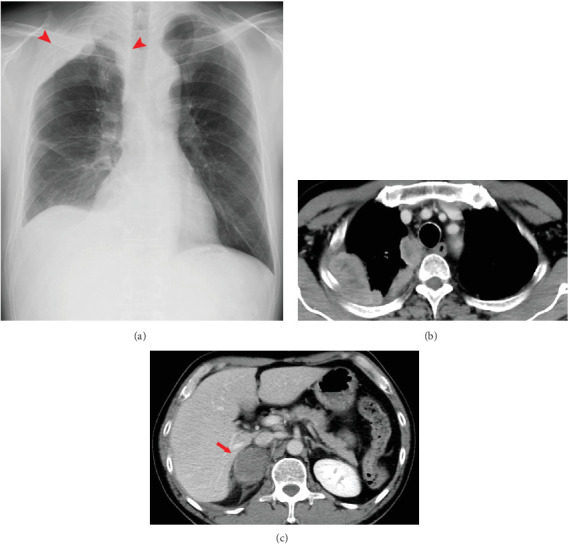
(a) Chest x-ray revealing right apical pleural thickening. (b) CT image demonstrating right continuous pleural thickening (arrowheads), including the mediastinal pleura and lymph nodes, (c) with a concurrent presence of right adrenal gland tumor (arrow). CT, computed tomography.

A 55-year-old male patient visited our hospital with complaints of chest and back pain. Upon examination, the chest x-ray revealed pleural thickening and mediastinal enlargement. Chest and abdominal contrast-enhanced CT scans revealed multiple tumor lesions that extend from the right apical pleura to the mediastinal lymph nodes, as well as a tumor in the right adrenal gland (Figure 4). Transbronchial needle aspiration was conducted, confirming the MPM (cT4N1M1 Stage IV) diagnosis. First-line treatment with nivolumab (240 mg every 2 weeks) plus ipilimumab (1 mg/kg every 6 weeks) was initiated.

Shortly after treatment initiation, pleural effusion and tumor enlargement rapidly progressed. These changes were considered pseudoprogression; thus, ICI treatment was continued with frequent drainage of pleural fluid by thoracentesis. Gradual improvement was found, and pleural effusions no longer increased without thoracentesis after approximately 2 weeks, and a significant reduction in tumor size and an almost complete resolution of pleural effusion were found after the first treatment cycle. However, interstitial pneumonia (Grade 1) developed at the end of the first treatment cycle, requiring a 1-month treatment interruption.

The second treatment cycle was resumed because the interstitial pneumonia remained mild and did not progress, but the patient complained of headaches shortly thereafter. Head MRI revealed pituitary gland enlargement (irAE hypophysitis) and no obvious brain tumor invasion findings. A lumbar puncture was performed and revealed increased mononuclear cells in the cerebrospinal fluid, confirming the development of aseptic meningitis, including irAE meningitis. Blood tests revealed decreased thyroid-stimulating hormone and cortisol and increased free triiodothyronine (FT3) and free thyroxine (FT4) levels (irAE hyperthyroidism [Grade 1]). Low sodium values and increased eosinophils were also observed, and these findings were considered to be due to hypopituitarism associated with irAE hypophysitis.

The differential diagnosis for the headache was irAE meningitis, irAE hypophysitis, and carcinomatous meningitis. High-dose steroids were expected to be effective in all pathologies, and as in Case 1, we selected high-dose DEX treatment considering not only irAE but also carcinomatous meningitis [6, 7]. After oral DEX (6 mg/day) was initiated, headache symptoms quickly improved, and eosinophil count, hypopituitarism-related electrolyte abnormalities, and thyroid function demonstrated a trend toward improvement. A few days later, cytology results of the cerebrospinal fluid showed no malignant findings, and the cause of the headache was considered irAE meningitis (Grade 3) or irAE hypophysitis (Grade 3).

The DEX level gradually tapered over 1 month. FT3 and FT4 decreased during the treatment, warranting additional levothyroxine (hypothyroidism [Grade 2]) administration.

**Figure 5:**
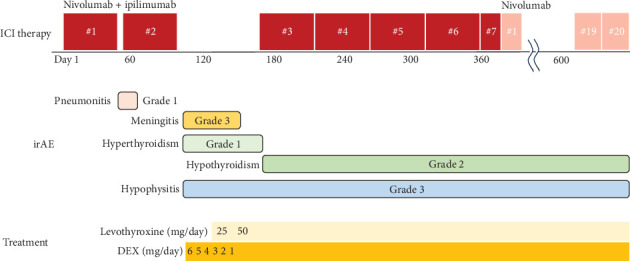
The summary of ICI therapy, irAE, and irAE-related treatment in Case 2. ICI, immune checkpoint inhibitor; irAE, immune-related adverse event; DEX, dexamethasone.

**Figure 6:**
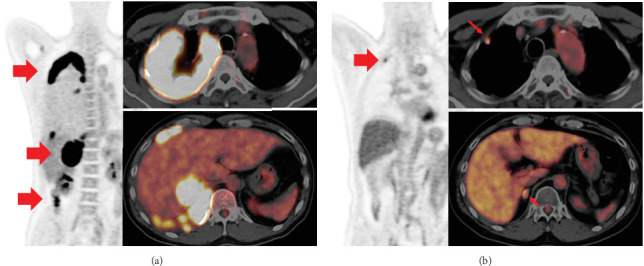
PET-CT scan (a) before ICI therapy and (b) after four treatment cycles. The patient has achieved partial response (PR). ICI, immune checkpoint inhibitor.

ICI therapy was repeatedly interrupted, and steroids were required to control for multiple irAEs in this case.  Figure 5 shows the progress of the initial four cycles when irAE was extreme. PET-CT scan after four cycles exhibited that the tumor had markedly shrunk, with only a small amount of residual uptake (Figure 6), and was judged as a partial response (PR).

**Figure 7:**
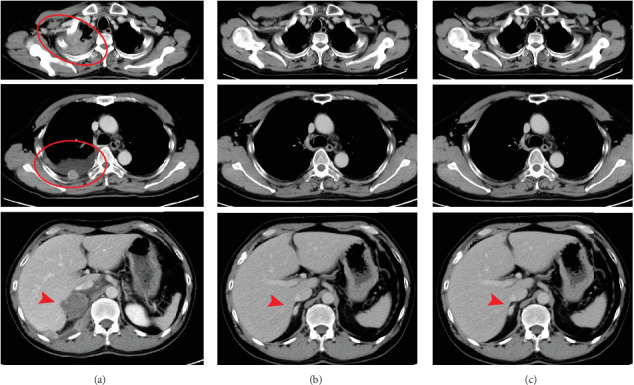
CT image (a) before ICI therapy and (b) after 9 months and (c) 20 months. The target lesions were widely spread as a large mass within the right pleura (circle) and adrenal gland (arrowhead) before therapy but are now localized to the metastatic site within the adrenal gland. CT, computed tomography; ICI, immune checkpoint inhibitor.

The side effects of irAE completely disappeared with the maintenance of small steroid and levothyroxine doses from the third course, and ipilimumab and nivolumab could be continued without discontinuation. However, a Phase III NIPPON study about the efficacy of platinum-based combination chemotherapy together with nivolumab plus ipilimumab in non–small cell lung carcinoma (NSCLC) reported many irAE-related deaths during the seventh treatment course, and the study was discontinued [8]. Considering the results of this study and the fact that this patient had already experienced multiple severe irAEs, we switched to nivolumab monotherapy after seven courses. However, the patient experienced no progression with only a tiny nodule in the right adrenal gland remaining after 20 courses of nivolumab monotherapy (Figure 7).

## 3. Discussion

ICI blocks immune-suppressive molecules that target the tumor. Adverse events associated with these agents occur through mechanisms distinct from conventional chemotherapy and are referred to as irAEs. The mechanisms underlying irAE development have multiple pathways, with common molecules involved in both tumor immunity and autoimmune processes. Therefore, a correlation of irAE occurrence with the treatment efficacy has been indicated [9] and has been reported in various cancer types [10–19] (Table 1). Not only the presence of irAEs but also the number [10, 18, 19] and severity [4] of affected organs have been implicated to correlate with prognosis in other malignancies.

To the best of our knowledge, no report has specifically assessed these associations in MPM, and this is one of few studies that indicate a positive association of irAE with better prognosis in patients with MPM. Conversely, the CheckMate 743 trial has revealed a trend toward extended OS in the group experiencing ICI therapy discontinuation due to severe irAEs [20]. This indicates that developing multiple and severe irAEs may correlate with MPM prognosis as well as other malignancies.

In our cases, the patients demonstrated significant antitumor efficacy despite developing serious multiple irAEs. Regarding irAE development, Grade 3 events were experienced with three organs in Case 1, and two events occurred in Case 2. All of these Grade 3 irAEs in both cases required long-term steroid use. The overall incidence of any-grade irAEs in the CheckMate 743 trial was 80%, with a Grade 3 or higher development rate of 31%, of which 23% required ICI administration interruption [20]. Reportedly, most patients with multiple irAEs had two irAEs (*n* = 40, 78%) in a study involving 623 patients with NSCLC treated with ICI monotherapy or combination [18]. Noteworthily, both of our cases experienced more than two irAEs, spanning four organs during the treatment period. Regarding antitumor efficacy, Case 1 achieved CR, and Case 2 maintained PR, which is near CR with high antitumor efficacy. The objective response rate in the CheckMate 743 trial was 40%, with a CR rate of only 2% [3]. Our cases experienced relatively severe irAEs and obtained significant antitumor efficacy compared to previous studies (Table 2).

The combination therapy with nivolumab plus ipilimumab for unresectable MPM has demonstrated high efficacy compared to conventional chemotherapy or single ICI [21]. Some studies tried the combination with chemotherapy [22], and further progress in chemotherapy in MPM is expected. Conversely, further development is required in dealing with various side effects, including irAEs. Based on our experiences, simply discontinuing ICI treatments is not feasible, and making a balance between irAE control and cancer management is important.

## 4. Conclusions

We reveal a potential association between the occurrence, the number of events, and the severality of irAEs and antitumor efficacy in MPM. Additionally, we emphasize the importance of appropriately managing irAEs in treating MPM with ICI. Further accumulating case reports regarding the association between irAE and antitumor efficiency in MPM are expected in the future.

## Figures and Tables

**Table 1:** References regarding the relationship between irAE and the prognosis.

**Study**	**Summary of the paper**	**Cancer type (number of patients)**	**Survival endpoints between patients with and without irAEs**
Hata et al. [10]	Patients with irAEs tended to have better OS and PFS in NSCLC, GC, and MM	NSCLC (50)	OS (NR vs. 14.5 vs. 3.3 months^a^; multiple irAEs vs. no irAE, *p* = 0.016) PFS (11.8 vs. 9.0 vs. 2.1 months^a^; multiple irAEs vs. no irAE, *p* < 0.001)
		GC (43)	OS (15.9 vs. 5.4 vs. 3.9 months^a^; multiple irAEs vs. no irAE, not statistically significant) PFS (4.7 vs. 2.2 vs. 1.9 months^a^; multiple irAEs vs. no irAE, *p* = 0.047)
		MM (38)	OS (12.8 vs. 4.8 vs. 4.8 months^a^; multiple irAEs vs. no irAE, *p* = 0.016) PFS (3.6 vs. 2.8 vs. 1.9 months^a^; multiple irAEs vs. no irAE, *p* = 0.033)
Grangeon et al. [11]	Longer OS and PFS and better ORR when irAEs occurred in a population of patients with advanced NSCLC treated with ICIs	NSCLC (270)	OS (NR vs. 8.21 months; HR 0.29, 95% CI 0.18–0.46, *p* < 0.001) PFS (5.2 vs. 1.97 months; HR 0.42, 95% CI 0.32–0.57, *p* < 0.001) ORR (212.9% vs. 5.7%; odds ratio 4.9, 95% CI 2.18–11.05, *p* < 0.001)
Nukaya et al. [12]	The development of an irAE correlated with both an improved median OS as well as median PFS in patients with mRCC treated with nivolumab and ipilimumab	MRCC (69)	Patients with vs. without irAEs had significantly prolonged OS (*p* = 0.012) and PFS (*p* = 0.002) No exact median OS is listed in this article
Maher et al. [13]	Patients who responded to treatment with an anti-PD-1/L1 antibody were more likely to report a related imAE	UCC (1747)	OS (HR 0.53, 95% CI 0.43–0.66); no *p* value listed
Das et al. [14]	GI cancer patients who experienced irAEs while on ICIs had marked improvements in PFS and OS compared to those who did not	GI (76)	OS (NR vs. 7.4 months, HR 0.11, 95% CI 0.03–0.36, *p* < 0.001) PFS (NR vs. 3.9 months, HR 0.13, 95% CI 0.05–0.3, *p* < 0.001)
Masuda et al. [15]	Development of irAEs was associated with clinical benefits for advanced gastric cancer	Gastric (65)	OS (16.8 vs. 3.2 months; HR 0.17, *p* < 0.001) PFS (7.5 vs. 1.4 months; HR 0.11, *p* < 0.001)
Foster et al. [16]	Developing irAEs is associated with superior ORR, PFS, and, in particular, OS of head and neck cancer patients	HNSCC (114)	OS (12.5 vs. 6.8 months, *p* = 0.0007) PFS (6.9 vs. 2.1 months, *p* = 0.0004) ORR (30.6% vs. 12.3%, *p* = 0.02)
Haratani et al. [17]	Development of irAEs was associated with OS and PFS in NSCLC	NSCLC (134)	OS (not reached vs. 11.1 months, *p* = 0.01; multivariable HR 0.285, 95% CI 0.102–0.675, *p* = 0.003) PFS (9.2 vs. 4.8 months, *p* = 0.04; multivariable HR 0.542, 95% CI 0.295–0.971, *p* = 0.04)
Shankar et al. [18]	Patients with multiple irAEs demonstrated the best OS compared to single irAE and no irAE in NSCLC	NSCLC (623)	OS (21.8 vs. 12.3 vs. 8.7 months^a^; multiple irAEs vs. no irAE, aHR, 0.57; *p* = 0.005; single irAE vs. no irAE, aHR, 0.86; *p* = 0.26) PFS (10.9 vs. 5.1 vs. 2.8 months^a^; multiple irAEs vs. no irAE, aHR, 0.39; *p* < 0.001; single irAE vs. no irAE, aHR, 0.68; *p* < 0.001)
Freeman-Keller et al. [19]	Greater OS benefit noted in patients who reported three or more irAEs in MM	MM (112)	A statistically significant OS difference was noted among those experiencing any irAE vs. those without (*p* ≤ 0.001), and greater OS benefit was noted in patients who reported three or more events (*p* ≤ 0.001) with a log-rank test. No exact median OS is listed in this article

Abbreviations: 95% CI, 95% confidence interval; aHR, adjusted hazard ratio; GC, gastric cancer; GI, gastrointestinal; HNSCC, head and neck squamous cell carcinoma; HR, hazard ratio; imAE, immune-mediated adverse event; irAEs, immune-related adverse events; MM, malignant melanoma; mRCC, metastatic renal cell carcinoma; NR, not reached; NSCLC, non–small cell lung cancer; OR, odds ratio; ORR, objective response rate; OS, overall survival; PFS, progression-free survival; TTNT, time to next treatment; UCC, urothelial cell carcinoma.

^a^Multiple irAEs versus single irAE versus no irAE.

**Table 2:** Comparison table of treatment efficacy and patterns of irAE onset in previous reports and our experimental cases.

	**CheckMate 743 [** 3, 20**]**		**Case 1**	**Case 2**
Antitumor effects	ORR CR PR Median PFS Median OS Median DOR	40 (%) 2 (%) 37~38 (%) 6.8 (months) 18.1~21.8 (months) 11.0~11.6 (months)	Maintain CR for over 12 months	Maintain PR for over 20 months
irAE	Any-grade Grade 3 or higher Need steroids	80 (%) 30~31 (%) 20 (%)	Colitis (Grade 3) Meningitis (Grade 3) Hepatitis (Grade 3) Renal dysfunction (Grade 2) Dermatitis (Grade 1)	Meningitis (Grade 3) Hyperthyroidism (Grade 1) Hypothyroidism (Grade 2) Hypophysitis (Grade 3) Pneumonitis (Grade 1)

Abbreviations: CR, complete response; DOR, duration of response; irAE, immune-related adverse event; ORR, objective response rate; OS, overall survival; PFS, progression-free survival; PR, partial response.

## Data Availability

The data that support the findings of this study are available on request from the corresponding author. The data are not publicly available due to privacy or ethical restrictions.
